# Therapeutical Treatment of Dental Pulps

**Published:** 1878-10

**Authors:** A. F. McLain

**Affiliations:** New Orleans


### THERAPEUTICAL TREATMENT OF DENTAL PULPS.

BY DR. A. F. M'LAIN, NEW ORLEANS.

*Read before the Southern Dental Association.*

*Continued from page 385.*

In treating the consequences of dead pulps, the therapeutical indications are obviously to remove them when it is possible, before their decomposition has commenced. If in the inflammatory stage, it should be subdued by the introduction of anodynes and sedatives. These consist of aconite, chloroform, creasote, or carbolic acid, morphine, etc., etc. When the inflammation has reached the peridental membrane, an application to the gum of equal parts of the tinctures of aconite and iodine will frequently suffice to remove the difficulty. Should this fail, resort may be had to blistering about the region of the tooth; which is done by means of cantharidal colodion, or with strong ammonia; but when the hyperæmia has assumed the congestive form, then leeching the gums may be efficacious. This mode of abstracting blood should not be adopted, however, at the inception of the inflammatory stage, as an encouragement to the afflux of blood to the part, already too great would be given, and thus aggravate the symptoms. The existence for some hours consecutively of such a state, the practitioner may conclude with certainty that the inflammation has passed into congestion.

Warmth and stimulants, within proper bounds be it understood, are most valuable therapeutical agents in the treatment of congestions. They act by expanding the arterial trunks, thus giving an impetus that sweeps back into the circulation the stagnant blood, which had been arrested in the veins and capillaries. On the other hand, cold applications frequently renewed, are of great benefit, as by their constringing effect on the vessels a determination of blood is moderated, thus allowing the circulation in the part to regain its equilibrium, by which means a resolution of an incipient inflammation is promoted. Heat and cold are merely relative terms; they are both sedative and stimulant, but inversely. To gain a sedative effect from cold, the application of it must be persistently continuous, which is obtained by frequent repetition, else the blood that has been repelled at first by the contraction of the vessels surges back, hence, instead of acting as a sedative it becomes a stimulant, which increases the local hyperæmia. It is, therefore, very evident that cold can be made either stimulating through its intensity and transitory application, or sedative by a persistent, non-intermitting course in its use.

Should the inflammatory congestion show signs of resolving into an abscess, the abstraction of blood becomes an immediate necessity. Leeching is preferable to scarification, as the lancet does not deplete sufficiently, besides increasing the irritation through the wound it makes. On the formation of an abscess, however, a free incision should be made into it, in order to relieve the distension and consequent pressure caused by the exudation that has taken place in the parts. At the same time, the pulp chamber and root canals require to be thoroughly cleared of all decomposed or decomposable matters, such as the blood-pulp, pus, or any other foreign irritating substances that may be found therein; after which the cavity ought to be well syringed out with tepid water, and a dressing of chloroform, aconite, iodine (*aqueous solution*) and carbolic acid or creasote, introduced on a lock of cotton loosely placed in the pulp cavity. This is particularly essential if pain exists, as by sealing the cavity the secretions and gases that might form in the tooth would find no escape, and in that way increase the pressure and consequently also the pain. Otherwise, a covering may be placed over the application, in order to prevent the remedies from being too much diluted or washed out. These dressings are to be renewed at first from day to day, then every other day, and finally once or twice a week until the abnormal symptoms have disappeared. An obstinate abscess may require from ten days to several months to be effectually subdued. A sinuous opening existing in the gum near the root of an affected tooth, the preparatory treatment need not be continued more than a few days, long enough to cleanse the pulp chamber and root canals of all extraneous matters and otherwise disinfect them; but a permanent filling may be introduced with little risk, as the vent thus afforded for the escape of the pus or gas that might be generated would prevent the natural consequences usually resulting from pent-up fluids. The remedies introduced into a tooth with an abscess may be a little more concentrated than those applied to exposed pulps or sensitive dentine; but it is well to reiterate here that the use of pure creasote or carbolic acid in a tooth in any case should be deprecated, particularly forcing it through the apical foramen as too many do in treating teeth. The escharotic nature of these agents can not act otherwise than excoriate the already diseased peridental membrane. Sometimes the introduction of carvacrol in connection with the previously named anodynes into an abscessed tooth will cause the secretion of pus to cease in a few days. Occasionally much good may be derived from varying the remedies, by substituting tincture of belladonna, essence of peppermint, or tincture of camphor, in lieu of carbolic acid and creasote. Again, camphor-chloral in the place of those articles just enumerated, but in a diluted state, be it understood.

When the discharge is persistent, failing to respond to the preceding modes of medication, diluted aromatic sulphuric acid, introduced on a probe armed with cotton lint through the tooth, acts very efficiently at times. The same agent may be tried at the apex of a root, by means of a perforation made through the gum and process. The great dependence placed by some operators on this method of curing alveolar abscesses, your essayist is free to confess has not availed him to that extent which its advocates claim for it. Chloride of zinc can be used in the same way, and by injection through the pulp cavity for indolent abscesses, with the view of breaking up the pyogenic membrane which lines the sac of the abscess, and also as a stimulalant for the promotion of a healthy action. Nitrate of silver might perhaps be used with equal advantage for such purposes, were it not for the objectionable and inevitable coloration of the tooth it produces when so employed. Surgeons, it is well known, are in the habit of curing most intractable caries of the bones with this agent. At all events, nitrate of silver, as well as sulphuric acid and chloride of zinc, may be used in the treatment of alveolar abscesses without incurring the risk of coloring a tooth, by introducing it to the apex of a root through an artificial or a fistulous opening.

An abscess which does not respond readily to the appropriate treatment is sometimes thoroughly healed by placing in the cavity a dressing well medicated with anodynes and antiseptics, and afterwards inserting a temporary filling, which may be left in for several weeks, and even for months, for the purpose of allowing the recuperative forces to have full play. If, after a length of time, no odor or any secretion appears on opening the cavity, a permanent filling may be made with little danger of subsequent bad results.

The remedial measures to be employed in a fully established EXODONTOSIS are *nil* as far as the author's experience goes. Prevention, if possible, would seem to be the only rational course to be pursued; and as this pathological condition often arises from a sub-acute form of peridental inflammation, or sometimes from a rheumatic predisposition to peridental irritation which excites hypertrophic action, hence the adoption of prompt antiphlogistic measures, such as leeching, blistering, and discutients, early in its symptoms, the disease might perhaps be prevented or cured. Rheumatic subjects being those most liable to this form of hypertrophy, a natural inference may be drawn, that when this affection is suspected, particularly in one having a constitutional tendency to rheumatism, the treatment proper for this last would also be good for EXODONTOSIS.

For NECROSIS of the roots of teeth, they having become foreign bodies through loss of vitality, their removal becomes prime necessity.

